# Left Ventricular Function during Acute High-Altitude Exposure in a Large Group of Healthy Young Chinese Men

**DOI:** 10.1371/journal.pone.0116936

**Published:** 2015-01-28

**Authors:** Mingyue Rao, Jiabei Li, Jun Qin, Jihang Zhang, Xubin Gao, Shiyong Yu, Jie Yu, Guozhu Chen, Baida Xu, Huijie Li, Rongsheng Rao, Lan Huang, Jun Jin

**Affiliations:** 1 Institute of Cardiovascular Diseases of PLA, Third Military Medical University, Chongqing 400037, China; 2 Department of Cardiology, Xinqiao Hospital, Third Military Medical University, Chongqing 400037, China; 3 Department of Medical Ultrasonics, Xinqiao Hospital, Third Military Medical University, Chongqing 400037, China; University Heart Center, GERMANY

## Abstract

**Objective:**

The purpose of this study was to observe left ventricular function during acute high-altitude exposure in a large group of healthy young males.

**Methods:**

A prospective trial was conducted in Szechwan and Tibet from June to August, 2012. By Doppler echocardiography, left ventricular function was examined in 139 healthy young Chinese men at sea level; within 24 hours after arrival in Lhasa, Tibet, at 3700 m; and on day 7 following an ascent to Yangbajing at 4400 m after 7 days of acclimatization at 3700 m. The resting oxygen saturation (SaO2), heart rate (HR) and blood pressure (BP) were also measured at the above mentioned three time points.

**Results:**

Within 24 hours of arrival at 3700 m, the HR, ejection fraction (EF), fractional shortening (FS), stroke volume (SV), cardiac output (CO), and left ventricular (LV) Tei index were significantly increased, but the LV end-systolic dimension (ESD), end-systolic volume (ESV), SaO2, E/A ratio, and ejection time (ET) were significantly decreased compared to the baseline levels in all subjects. On day 7 at 4400 m, the SV and CO were significantly decreased; the EF and FS Tei were not decreased compared with the values at 3700 m; the HR was further elevated; and the SaO2, ESV, ESD, and ET were further reduced. Additionally, the E/A ratio was significantly increased on day 7 but was still lower than it was at low altitude.

**Conclusion:**

Upon acute high-altitude exposure, left ventricular systolic function was elevated with increased stroke volume, but diastolic function was decreased in healthy young males. With higher altitude exposure and prolonged acclimatization, the left ventricular systolic function was preserved with reduced stroke volume and improved diastolic function.

## Introduction

In recent years, increasing numbers of individuals have been exposed to high-altitude environments through entertainment, physical training, work, or habitation. Atmospheric pressure falls with ascending altitudes, and with it, there are decreases in the partial pressure of inspired oxygen, arterial pressure of oxygen and arterial oxygen saturation. Therefore, exposure to hypobaric hypoxia is associated with a series of adaptive responses, such as cardiovascular compensation, to combat the diminished arterial oxygen content. The initial cardiac response to hypoxia is characterized by an increase in cardiac output with tachycardia [1–3], which helps maintain oxygen delivery. Accurate delineation of the cardiovascular alterations after subjects have been at high altitudes has interested investigators since the early years of the last century [4,5]. However, hostile environmental conditions, sample size limitations and taking place solely in simulative altitude chambers [6,7], where the environment and activity are continuously controlled, lacking of cold, wind field environments have been the main deterrents, so the effect of acute high-altitude exposure upon left ventricular stroke volume is a controversial problem. Previous reports show stroke volume adaptations ranging from a decrease to a distinct increase [6,8–10].

In addition, the convenience of current transportation methods allows for rapid ascent to altitudes that can compromise acclimatization and expose inexperienced climbers to the hazards of high altitude. Thus, it is urgent to identify the pattern of left ventricular function changes in high-altitude fields. We therefore assessed left ventricular function by conventional Doppler method analysis in a large group of healthy non-acclimatized young males at 500 m, within 24 h after rapid ascent by train to 3700 m and on the 7^th^ day of acclimatization at 4400 m.

## Materials and Methods

### Subjects

In total, 139 healthy young Chinese males who lived in the low lands (500 m) were recruited for the study. The average age, height, weight, body mass index (BMI) and body surface area (BSA) at the time of present study were 22.2±3.3 years old (y)(ranged from 18 to 33 years old), 171.4±4.5 centimeters, 63.4±7.1 kilograms, 21.4±2.0 kilograms/meters^2^(Kg/m^2^) and 1.7±0.1 square meters(m^2^), respectively. Subjects with any one of the following conditions were excluded: respiratory diseases, cardiovascular diseases, malignant tumors, liver and kidney dysfunction and diseases of the immune system, as well as people with psychiatric disorders who could not complete the questionnaires. Thirty-two of the 139 subjects had altitude exposure history from six months ago, but no high-altitude cerebral edema or pulmonary edema had occurred. Seventy-five of them were cigarette smokers. In addition, the subjects did not take medication or receive any intervention before ascending.

The study was approved by the Ethics Committee of Xinqiao Hospital, the Second Clinic Medical College of the Third Military Medical University of the PRC. Written informed consent was obtained from all participants after being informed of the study’s purpose, procedures, and inherent risks and benefits.

### Study protocol

**Fig 1:**
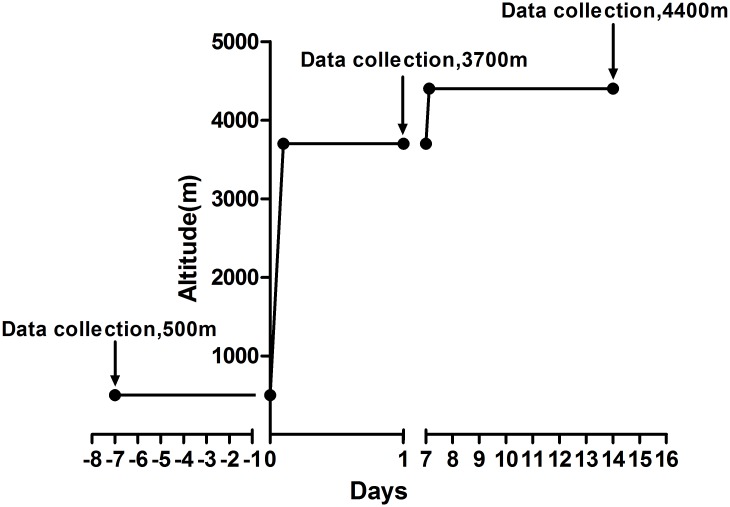
Altitude ascent profile of participants from plain to plateau. All subjects ascended to 3700 m (Lhasa in Tibet) in 2.5 hours by plane from 500 m (Chengdu in Sichuan province). After they acclimatized at 3700 m for a week, the subjects traveled by motorcar and arrived in Yangbajing (in Tibet, at 4400 m) within 3 hours; they in Yangbajing for 7 days. For all participants, the starting data collection point was in Chengdu (500 m), the second data collection point was in Lhasa (3700 m) within 24 h of arrival, and the third data collection was on the 7^th^ day in Yangbajing (4400 m).

All subjects ascended to 3700 m (Lhasa in Tibet) in 2.5 hours by plane from 500 m (Chengdu in Sichuan province). The subjects traveled by motorcar and arrived in Yangbajing (in Tibet, at 4400 m) within 3 hours, where they then stayed for 7 days after they had acclimatized at 3700 m for a week (Fig. 1). Structured case report form (CRF) questionnaires were used to record demographic data (age, height, weight, and ethnicity), physiological data (BP, SaO_2_ and HR), previous high-altitude exposure (>2500 m) before this travel, and symptoms related to AMS (headache, dizziness/lightheadedness, gastrointestinal symptoms, insomnia and fatigue/weakness).

All of the above mentioned procedures were performed at 500 m within one week before ascension to Chengdu, within 24 h after arrival at 3700 m in Lhasa at approximately 13:00 pm June 23^rd^–25^th^, 2012 (examinations were performed at approximately 8:00 am–12:00 pm the next morning upon arrival), and on the 7^th^ day after ascending to 4400 m (Yangbajing, July 7^th^–9^th^, 2012, following 7 days of acclimatization at altitudes of 3700 m in Lhasa). Heart rate, oxygen saturation, and conventional transthoracic Doppler echocardiographic measurements were performed at the above-mentioned three time points. All of the questionnaires and measurements were non-invasive.

### AMS

Acute mountain sickness (AMS) is commonly experienced by people traveling to high altitudes, typically those above 2500 m. The AMS diagnoses were based on the Lake Louise score (LLS), an international standard scoring system for AMS [11]. This defines the syndrome of AMS as characterized by five self-reported symptoms: headache, dizziness or lightheadedness, gastrointestinal symptoms (anorexia, nausea or vomiting), difficulty sleeping, and fatigue or weakness. Every item is measured on a 4-point Likert scale, range from 0 to 3, with 0 indicating none, 1 for slight, 2 for moderate, and 3 for severe. The AMS, defined as the total score of the 5 items, must amount to a 3 or higher, but headache must be present in the context of these symptoms within several hours or days of arrival at altitude. A total score of 3–4 is defined as mild AMS, and a total score of 5 or higher is defined as severe AMS [12,13].

### Physiological data

The oxygen saturation (SaO_2_) was measured with a pocket pulse oximeter (NONIN-9550, Nonin Onyx, America). The blood pressure (BP) and heart rate (HR) were determined with a sphygmomanometer (HEM-6200, Omron, China). These data were measured in triplicate after the subjects had rested in a seated position for 20 minutes. The mean arterial pressure (MAP) was calculated according to the systolic blood pressure (SBP) and diastolic blood pressure (DBP): MAP = DBP+ (SBP—DBP)/3.

### Measurement of left ventricular systolic function

A color Doppler echocardiography instrument (CX 50, Philips Ultrasound System, Andover, MA, USA) with a probe of 2 to 4 MHz was used to measure the left ventricular end-diastolic dimension (LVEDD), end-systolic dimension (LVESD) and HR, where the left parasternal long-axis and the short-axis views at the mid-left ventricular level were obtained when the heart rate was in a steady state. The left ventricular end-systolic volume (LVESV) and left ventricular end-diastolic volume (LVEDV) were obtained by Teichholz’s formula [14]. The stroke volume (SV), cardiac output (CO), ejection fraction (EF), and fractional shortening (FS) were also calculated. The stroke volume index (SVI) and cardiac index (CI) were the SV and CO, adjusted by BSA, respectively.

### Left ventricular diastolic function and Tei index

**Fig 2:**
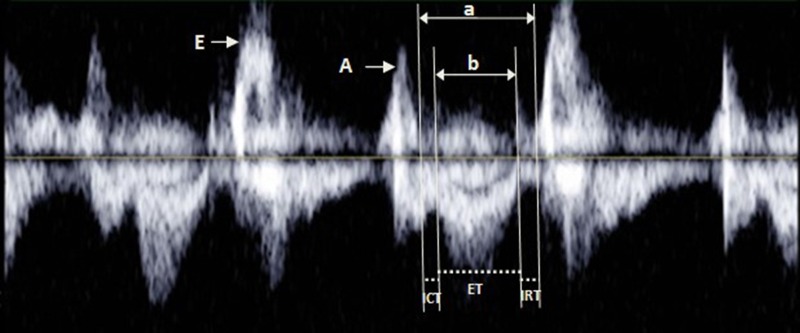
Illustration of a pulsed Doppler diagram showing the measurement of each parameter for calculating the Tei index. a = isovolumetric contraction time (ICT) + ejection time (ET) + isovolumetric relaxation time (IRT); b = ET. Tei index = (ICT+IRT)/ET = (a-b)/b.

In the Doppler examinations, the mitral inflow velocity pattern was recorded from the apical long-axis view, and the pulsed wave Doppler sample volume was positioned at the tip of the mitral leaflet during LV diastole to measure the early (E) and late (A) diastolic wave peak velocities; the E/A ratio was obtained through dividing the E wave peak velocity by the A wave peak velocity. The LV outflow velocity pattern was recorded from the apical long-axis view, with the pulsed wave Doppler sample volume positioned just below the aortic valve. Then, the time intervals for calculating the LV Tei index were measured from the mitral inflow and the LV outflow recordings [15] (Fig. 2). Three consecutive beats were measured and averaged for each parameter. In Fig. 2, the time from the cessation to the onset of mitral inflow (a) represents the interval between the mitral valve closure and opening, which is equal to the sum of the isovolumic contraction time (ICT), ejection time (ET) and isovolumic relaxation time (IRT). The ET (b) of the LV was measured in the LV outflow velocity pattern. The Tei index was defined as the sum of the ICT and IRT divided by the ET; thus, the LV Tei index was determined using (a-b)/b [16]. A single observer performed offline analysis of these parameters after returning to a low altitude.

### Statistical analysis

The continuous data are expressed as the mean ± SD. The baseline and the two high-altitude measurements in the lowlanders were analyzed using a one-way analysis of variance for repeated measures, followed by Newman-Keuls post hoc comparisons where appropriate. All data and calculations were performed with SPSS 19.0 software (Chicago, IL). A two-sided ±-level of significance of 0.05 was used for all tests, and a probability value less than 0.05 was considered to be statistically significant. Before analysis, all data were reviewed by the principal investigator for completeness.

## Results

### AMS

Within 24 hours after arrival at 3700 m, 75 of 139 subjects (53.96%) met the criteria for AMS; there were 43 mild AMS cases and 32 severe AMS cases. There were still 27 subjects (19.42%) who conformed to the Lake Louise score criteria after 7 days of acclimatization at an altitude of 4400 m, ten of whom had severe AMS, comparatively, and seventeen of whom had mild AMS. Fortunately, there was no one experienced high-altitude cerebral edema or pulmonary edema during this trip.

### Physiological parameters

As shown in Table 1, the SaO_2_ value was significantly reduced in all subjects from 98.42±x0.94% to 88.92±2.67% (P < 0.001) at resting state upon acute high-altitude exposure. However, the HR was significantly elevated from 62.47±8.98 beats/min to 82.91±11.56 beats/min (P < 0.001) under hypoxic conditions. However, the BP showed almost no change. After 7 days acclimatization at an altitude of 4400 m, the SaO_2_ value was further reduced from 88.92±2.67% to 87.96±2.57% (P < 0.01). However, the HR was further elevated from 82.91±11.56 beats/min to 86.85±15.55 beats/min (P < 0.01). The BP was increased after 7 days acclimatization at an altitude of 4400 m compared with the BP within 24 hours upon arrival at 3700 m, and the diastolic blood pressure (DBP) and mean arterial pressure (MAP) were significantly higher than those at sea level.

**Table 1:** Physiologic parameters at low and high altitude.

Physiologic parameters	500 m	3700 m, 24 h	4400 m, 7 d
SaO_2_ (%)	98.42±0.94	88.92±2.67**	87.96±2.57** ^‡^
HR (beats/min)	62.47±8.98	82.91±11.56**	86.85±15.55** ^‡^
SBP (mmHg)	116.14±11.67	114.56±10.64	117.45±11.44^**†**^
DBP (mmHg)	74.57±10.90	74.96±9.57	78.40±10.09** ^‡^
MAP (mmHg)	88.42±10.65	88.16±9.31	91.41±9.74* ^‡^

Normally distributed data are presented as the means ± standard deviations.

*P<0.05,

**P<0.01, compared with 500 m;

^†^P < 0.05,

^‡^P < 0.01, compared with 3700 m. SaO_2_, oxygen saturation; HR, heart rate; SBP, systolic blood pressure; DBP, diastolic blood pressure; MAP, mean arterial pressure.

### Left ventricular size

Comparisons were made between measurements at three different altitudes with respect to global left ventricular size (Table 2). Within 24 hours after arrival at 3700 m, the LVESD and LVESV were significantly decreased (P < 0.01), with the ESV especially reduced from 38.51±9.18 ml to 33.80±6.34 ml (P < 0.001). Furthermore, the LVEDD and LVEDV did not show significant alterations. After 7 days of acclimatization at an altitude of 4400 m, the LVESD and LVESV were further reduced compared with the corresponding values at 3700 m and were far lower than at sea level (P < 0.001). In addition, the LVEDD and LVEDV were also significantly decreased, and the LVEDV was reduced from 101.89±11.92 ml to 96.06±9.98 ml (P < 0.001).

**Table 2:** Left ventricular size and function at low and high altitude.

Item	500 m	3700 m, 24 h	4400 m, 7 d
Left ventricular size			
EDD (mm)	46.82±3.03	46.78±2.50	45.82±2.00** ^**‡**^
ESD (mm)	30.29±2.91	29.36±2.32**	28.75±1.81** ^**†**^
EDV (ml)	103.16±16.70	101.89±11.92	96.06±9.98** ^**‡**^
ESV (ml)	38.51±9.18	33.80±6.34**	31.80±4.77** ^**†**^
Left ventricular function			
EF (%)	62.87±5.61	66.90±4.18**	66.96±3.07**
FS (%)	35.41±4.72	37.26±3.55**	37.27±2.45**
SV (ml)	64.65±10.95	68.09±8.38**	64.27±6.86^**‡**^
SVI	38.06±6.54	40.27±5.61**	38.06±5.28^**‡**^
CO	4.04±0.84	5.38±1.07**	4.81±0.94** ^**‡**^
CI	2.38±0.50	3.17±0.66**	2.85±0.61** ^**‡**^

Normally distributed data are presented as the means ± standard deviations.

**P<0.01, compared with 500 m;

^†^P < 0.05,

^‡^P < 0.01, compared with 3700 m. EDD, end-diastolic dimension; ESD, end-systolic dimension; EDV, end-diastolic volume; ESV, end-systolic volume; EF, ejection fraction; FS, fractional shortening; SV, stroke volume; SVI, stroke volume index; CO, cardiac output; and CI, cardiac index.

### Left ventricular systolic function

Regarding the global LV systolic function, as expected, the EF, FS, CO, and CI increased significantly under hypoxic stress compared to the corresponding baseline values in all subjects (Table 2). However, in our study, the SV and SVI were significantly increased during hypoxic conditions compared to the same measurements under normoxia (P = 0.001).

After 7 days of acclimatization at a higher altitude (Yangbajing, 4400 m), the SV, SI, CO and CI decreased significantly compared to the corresponding measurements in Lhasa (3700 m) in all subjects (P < 0.01) (Table 2), but the CO and CI were still higher than those parameters at 500 m, and the SV and SI returned to baseline levels. There were no significant alterations after lower hypoxic stress in terms of EF and FS compared to the corresponding measurements in Lhasa (3700 m), but the EF and FS were significantly higher than the same measurements at sea level.

### Left ventricular diastolic function

**Fig 3:**
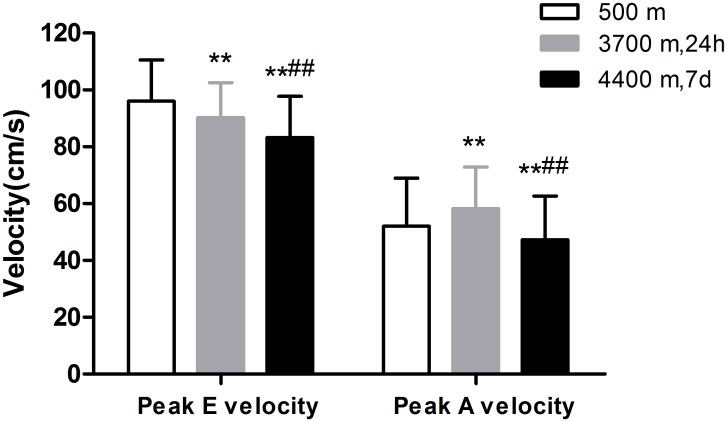
Comparison of mitral peak early diastolic filling velocity and peak late diastolic filling velocity in 139 lowlanders at the altitudes of 500 m, 3700 m and 4400 m. At the elevated altitudes, the peak E velocity decreased, initially increasing within 24 h at 3700 m but decreasing by day 7 at 4400 m. (Note: **P<0.01, compared with 500 m; ^**##**^P<0.01, compared with 3700 m.)

**Fig 4:**
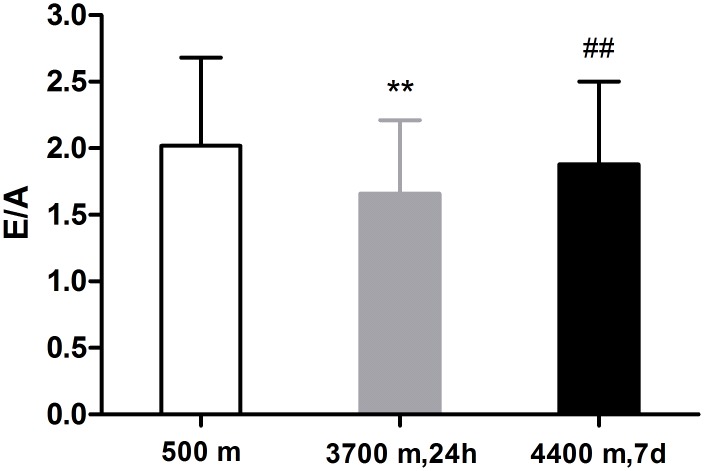
Comparison of the ratio of the early diastolic filling velocity to the late diastolic filling velocity (E/A ratio) in 139 lowlanders at the altitudes of 500 m, 3700 m and 4400 m. At the elevated altitudes, the E/A ratio decreased, though it increased on day 7 at 4400 m compared with the ratio observed at 3700 m. (Note: **P<0.01, compared with 500 m; ^**##**^P<0.01, compared with 3700 m.)

As shown in Fig. 3, within 24 h after acute high-altitude exposure, the mitral peak E velocity (early LV filling) decreased (96.03±14.55 cm/s vs. 90.26±12.28 cm/s, P = 0.001), the peak A velocity (filling due to auricular contraction) increased from 52.00±16.94 cm/s to 58.23±14.64 cm/s and the E/A ratio (an index of early—late LV filling) was significantly decreased (P < 0.001)(Fig. 4). At day 7 after acclimatization at 4400 m, both the mitral inflow peak E-velocity and peak A-velocity were significantly decreased compared with those values in Lhasa and in the lowlands, resulting in a slightly increased E/A ratio of the participants compared with that in Lhasa, which was still lower than that at sea level.

### LV Tei index

**Fig 5:**
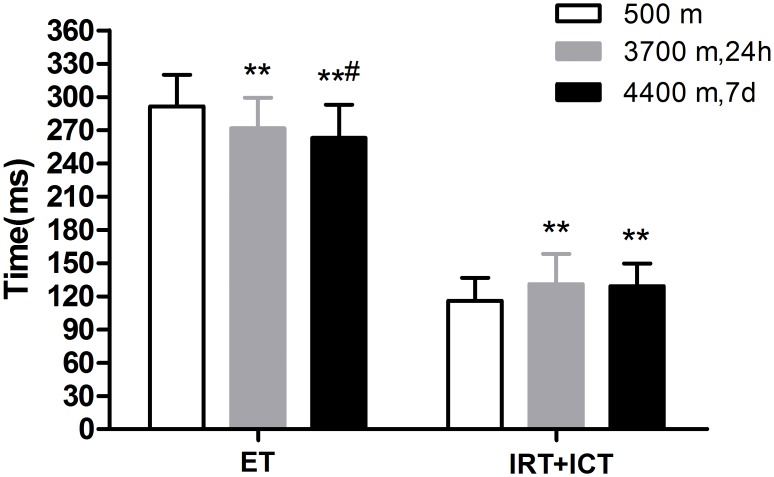
Comparison of the ejection time (ET) and the sum of isovolumic contraction time (ICT) plus isovolumic relaxation time (IRT) in 139 lowlanders at the altitudes of 500 m, 3700 m and 4400 m. At the elevated altitudes, the ET decreased, and it further decreased on day 7 at 4400 m compared with that at 3700 m. However, the sum of ICT and IRT was significantly prolonged at a plateau. (Note: **P<0.01, compared with 500 m; ^**#**^P<0.05, compared with 3700 m.)

**Fig 6:**
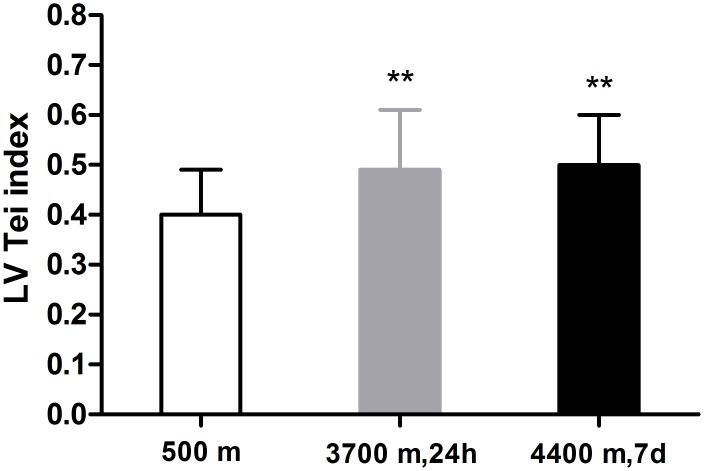
Comparison of the left ventricular Tei index (LV Tei index) in 139 lowlanders at the altitudes of 500 m, 3700 m and 4400 m. The LV Tei index was significantly increased at high altitude, but there was no significant difference between the values at 3700 m and 4400 m. (Note: **P<0.01, compared with 500 m.)

Fig. 6 shows the changing LV Tei index pattern at 500 m, within 24 h after acute 3700 m high-altitude exposure and day 7 after acclimatization at 4400 m. Within 24 h after acute high-altitude exposure, due to a significant prolongation of the sum of the ICT and IRT from 115.85±20.68 ms to 130.84±27.37 ms (P < 0.01) (Fig. 5) and a significant shortening of the ET (291.53±28.54 ms vs. 271.74±27.46 ms, P < 0.001), the LV Tei index was significantly higher than that in the lowlands (0.40±0.09 vs. 0.49±0.12, P < 0.001). In addition, on day 7 after acclimatization at 4400 m, the LV Tei index was further increased, though not significantly (0.49±0.12 vs. 0.50±0.10, P > 0.05), along with a further decreased ET (from 271.74±27.46 ms to 262.94±30.01 ms, P < 0.05) and a preserved ICT and IRT sum (130.84±27.37 ms vs. 129.16±20.34 ms, P > 0.05).

## Discussion

Most investigations have reported that altitude-related hypoxia induces tachycardia [10,17], which is caused by an increase in sympathetic activity [18], as evidenced by elevated plasma and urinary norepinephrine and epinephrine concentrations [19,20]. We also confirmed this finding, and even with prolonged acclimatization, the HR is higher in the present study following high-altitude exposure. In addition, the blood pressure was unchanged upon acute high-altitude exposure, which is in keeping with previous observations [21]. With higher altitude exposure, the systolic blood pressure and diastolic blood pressure were elevated, which may also suggest that the activation of neurohumoral factors is stronger than the effects of hypoxia-induced vasodilatation.

In our study, the LV contractility was elevated during exposure to high altitude in all subjects. This is in keeping with previous findings that suggested that LV systolic function was improved in hypoxic healthy volunteers [1–3,22,23] and even in studies referring to adults and children exposed to high altitude [21,24]. The improved cardiac systolic function in hypoxic environments allows for the output of the maximum amount of blood to transfer a sufficient amount of oxygen to support the metabolic demands of a body challenged by hypoxic stress [9]. As expected, the present data did show an improvement in cardiac output in all subjects, due to their elevated HR and SV. Our findings revealed that the stroke volume was increased with unaltered left ventricular preload but marked LV ejection fraction and fractional shortening, leading to a significant decrease in the end-systolic volume. The reduction of stroke volume at high altitude has been reported many times before [7,10,25]. Unchanged stroke volumes at rest were observed between measurements from a hypobaric chamber to those in high-altitude fields [6,26]. The differences between these studies may be related to the limitations of the small sample size and due to their taking place solely in simulative chambers without true environment stimulation. Furthermore, hypoxia was found to be associated with a decreased early diastolic mitral filling velocity annuli E/A ratio, resulting from an increase in left atrial contraction. These results are in keeping with previous reports of hypoxic exposure-induced LV diastolic dysfunction according to transmitral inflow [2,3,27–29]. Cardiac contraction is an energetic process, and cardiac relaxation is far more energy consuming [30]. Thus, the cardiac diastolic dysfunction observed prior to systolic function in response to hypoxia in a hypobaric chamber [1,6] results from a rapid decrease in high-energy phosphate metabolism in the cardiac left ventricle in humans [31].

With acclimatization to higher altitudes, cardiac output tended to decrease with increased hemoglobin and a greater capacity for carrying oxygen to tissues. Furthermore, the stroke volume was obviously reduced because of significant decrease in LV preload in all subjects. There are two possible explanations for the decreased LV preload. The first would be LV relaxation dysfunction because of a reduced energy supply [32,33]. The second explanation relates to the decrease in plasma volume that has been observed in lowlanders at altitude in many studies [34–37]. Hypoxic exposure at a higher altitude has been associated with decreased mitral early and late inflow velocity and an unchanged annuli E/A ratio compared with sea level in all subjects. These changes in the LV filling patterns consistent with impaired LV relaxation at high altitude have been observed in previous echocardiographic studies [3,27,28].

The LV Tei index has been proven to be a sensitive indicator of the global left ventricle function [38]. In the present study, the LV Tei index was significantly increased due to ICT and IRT prolongation and ET shortening upon acute exposure to high altitude. ET shortening may be related to an increase in HR. After 7 days of acclimatization at a higher altitude, the LV Tei index was still obviously higher than at sea level, in accordance with a previous study [39] in which the LV Tei index was also significantly elevated after 50 d of high-altitude exposure. Yakabe et al. [40] discovered that the Tei index is preload-dependent, and Cheung et al. [41] revealed that the afterload increase, preload reduction, and HR [42] are associated with significant increases in the Tei index. In our study, the elevated LV Tei index may be attributed to a reduced left ventricular preload, higher SBP and higher HR in all subjects after 7 days of acclimatization at 4400 m.

### Limitations

Several limitations exist in our study. First, in this study, all of the subjects were young adult males, which makes it difficult to extend our findings to other populations. Second, tissue Doppler imaging (TDI) was not used in this study; instead, we performed M-mode and pulse Doppler echocardiographic examinations that are commonly used in the clinic. In future studies, TDI will be used, as it will provide a more comprehensive evaluation of cardiac function. Lastly, we did not examine changes in the blood volume, plasma volume, erythrocyte volume or urine volume in this study, and thus, the reason for the preload reduction is not clear.

## Conclusions

In conclusion, left ventricular contractility is elevated, but diastolic function was decreased during exposure to high altitude, which is associated with an improvement in the LV ejection fraction, fractional shortening and stroke volume in healthy young males. With higher altitude exposure and prolonged acclimatization, the left ventricular systolic function was preserved, and there was improved diastolic function, which is associated with a preserved LV ejection fraction and fractional shortening and an elevated E/A ratio but a decreased stroke volume in all subjects.
